# Stories Told Together: Male Narratives of Non-Monogamous Bi+ and Heterosexual Men

**DOI:** 10.1007/s10508-021-02008-6

**Published:** 2021-06-07

**Authors:** Aurelio Castro

**Affiliations:** Department of Medicine and Surgery, University of Bologna, Via Giuseppe Massarenti 9, 40138 Bologna, BO Italy

**Keywords:** Non-monogamies, Bisexuality, Masculinity, Narrative studies, Heterosexuality, Sexual orientation

## Abstract

The stories we tell about our identities and sexual orientations shape how we perform gendered scripts and negotiate relationships with significant others. Previous literature inquired the styles and outcomes of consensual non-monogamous (CNM) relationships, but more research is need on how CNM men resist or abide to hegemonic models of masculinity. To understand how constructions of masculinity and conceptualizations of sexual orientation are embedded in CNMs, the study analysed the stories of non-monogamous Bi+ and heterosexual men. Following a critical narrative approach, the study inquired the diverse conceptualizations of masculinity, sexual orientation and relationship practices in the narratives of 20 non-monogamous Bi+ and heterosexual identified men. The semi-structured in-depth narrative interviews (105 min on average) were analyzed via Nvivo 12 and explored their stories of desire and the sense-making process of being sexually oriented to one or more genders and to one or more partner/s. Engaging in non-monogamy was signified as a relevant insight from their personal stories and/or from adopting new concepts of desire beyond the “love as a zero-sum game.” The latter theme was also shared by many heterosexual participants that, when negotiating a non-monogamous agreement, signified their attractions to more than one person as part of their personal identity. Finally, the paper discusses how non-monogamous spaces can offer a positive and safe space for bisexuals/Bi+ people to explore and reaffirm their identities, constantly challenged by biphobia, invisibility, and erasure. Experiences and stories of Italian cisgender Bi+ and heterosexual men cannot be generalized to the whole spectrum of masculinities within CNM spaces, and the study lacks how other gendered and sexual subjectivities construct masculinity. Diverse stories and construction of sexuality and gender can lead to similar relationship preferences and understanding how we signify them can greatly improve our understanding of intimacies.

## Introduction

During the last years, a growing number of studies inquired what kind of relationships exists outside the normative borders of monogamy and how non-monogamous people live and signify them (Barker & Langdridge, 2010). The present research expands this trend by discussing how the type of stories we tell about our sexual orientation and masculinity—individually and within general society—affects the relational sense-making of non-monogamous men.

Using critical narrative interviews (Emerson & Frosh, 2004; Riessman, 1993), the paper inquires how Bi+ and heterosexual men interweave gender and sexual orientation into their stories as sexually oriented men (Diamond, 2008). The research aims to voice and recognize the complexities of male stories, which traditional models of sexuality construct as monolithic, immutable and essentially biological in their performances (Ferrero-Camoletto & Bertone, 2010; Janssen et al., 2008). Heteronormative and monosexual accounts of sexuality have historically erased the experiences and stories of non-exclusive individuals and identities (Barker & Langdridge, 2008), fetishizing women’s bisexual orientation (Pond & Farvid, 2017) and denying the existence of non-exclusivity in men and male bisexualities (Rosenthal et al., 2012).

Within the present research, sexual orientation is understood as a consistent and enduring pattern of desire towards one gender, another or more than one (Barker et al., 2012), regardless of the degree of those attractions and if desires are acted or not with sexual behavior (Diamond, 2008). As sexual studies showed, there are different components of sexual orientation (e.g., desire, action/behavior, fantasies, identity and more) which are independent from each other, giving us the potential of experiencing non-exclusive desires in many different and valid ways (Diamond, 2008; Savin-Williams, 2006) and to more than one person (Manley et al., 2015). Desires and sexual identities can be flexible and change over time (Diamond, 2016; Katz-Wise & Hyde, 2015); in fact, non-exclusive individuals are the majority within LGBTQIA + people (Diamond, 2016) and are present for heterosexuals as well (Savin-Williams & Vrangalova, 2013). Similar to how traditional model of sexual orientation as a “compass”—firmly crystallized for the rest of a person’s life—are not efficient to understand the diverse experiences of sexuality (Diamond, 2012), it is not surprising that the socially constructed idea of monogamy might not be the only and best form of relationship.

### Monogamies and Non-Monogamies

Similar to hegemonic models of sexual orientation, relationships between individuals have been historically constructed to exclude and erase diversity to preserve hierarchies in society (Klesse, 2007); the pervasive norm of monogamy does not reflect the whole experience of human relationships and must be deconstructed. As written by Barker (2005), the dominant construction of sexuality assumes that relationships must be: between a man and a woman, in a monogamous relationship and with the man taking the active role in it, whereas women must be passive (Barker, 2005). Consensual non-monogamies or CNM (Veaux & Rickert, 2014) challenge the assumption of mandatory monogamy by including diverse understanding of relationships in contrast to a single hegemonic agreement. Under the CNM umbrella we find, instead, a set of relational, sexual and/or romantic agreements or styles outside of the societal assumption that relationships “must” be exclusive, that extra-dyadic desires or sex cannot be consensual, and finally, that monogamy is the only ethical and possible way of being in a relationships (Barker, 2010; Gusmano, 2019a; Moors et al., 2017).

Within consensual non-monogamies (or CNM), all the people involved, directly or indirectly, in the relationship are aware and/or agree to “navigate” relationships as non-exclusive between two or more partners; non-monogamous also refers the identity that people who engage in non-monogamy might adopt to describe themselves and not just their relationships (Barker, 2010). By conceptualizing all relationships agreements as “rules” accepted by partners, whether they are discussed or not (Moors et al., 2017), within monogamy these rules assume that two partners in a relationship must be exclusively involved both sexually and emotionally in the relationship (Moors et al., 2017). Within consensual non-monogamies, there is generally more relationship variability, and given the variety of experiences and potential agreements between people, it is difficult to provide unambiguous and comprehensive definitions of the diverse forms of CNM like polyamory, relational anarchy, swinging or otherwise (Gusmano, 2019a). The principle of consent is fundamental for CNM since they are based on honesty, commitment, integrity, equity, respectful negotiation and decision making (Anapol, 2010). Consent is the focal points of CNM and stands in contrast (Moors et al., 2017) to infidelity (e.g., having other sexual or romantic relationships without the consent of all partners), although also CNM are not exempt from cheating. One of the widespread forms of CNM that gained a lot of visibility even in mainstream media is polyamory, defined by Veaux and Rickert (2014), as “the state or practice of maintaining multiple sexual and/or romantic relationships simultaneously, with the full knowledge and consent of all the people involved.” Among others, the most frequent forms of non-monogamous relational agreements are: triads (three people involved with each other), quads, polyamory, swinging, open relationships, relational anarchy (Nordgren, 2006) and “monogamish” relations (monogamous couples who have threesomes due to transgression; Savage, 2012). Overall, under the umbrella of non-monogamies the partners involved agree to potentially entertain in multiple romantic and/or sexual relationships simultaneously (Conley et al., 2013); finally, non-monogamous relationships are not necessarily sexual or romantic; therefore, many asexualities also navigate the CNM umbrella (Scherrer, 2010). In CNM, not all partners necessarily have multiple relationships at the same time (e.g., only one person engages with other people), negotiating and discussing the “agreements or rules” is huge part of these relationships (e.g., discussing polyfidelity), which ensures transparency and to make explicit/anticipate the multiple outcomes within the relationship and the parties involved (Klesse, 2007). Contrary to mononormative assumptions, studies showed that having multiple partners/relationships to satisfy different needs does not affect the quality of relationships, since they are independent from one another, and that non-monogamous people do not seek other relationships due to little satisfaction in theirs (Mitchell et al., 2014; Moors et al., 2017).

### Contextualizing Non-Monogamies and Bisexualities in Italy

Concerning CNM, a growing number of studies provided useful insights and reflections for the Italian context (Braida, 2020; Grande & Pes, 2018; Gusmano, 2019a, 2019b); although there is currently no option available in Italy for multiple relationship recognition (Palazzo, 2018), CNM communities in Italy have diverse online spaces for discussing issues and sharing strategies and, finally, until 2019 a national meeting called OpenCon. Gusmano (2019b) identified two types of activist approaches to consensual non-monogamies in Italy: an experiential approach that focuses on sharing experiences and community building and a radical approach focused on addressing “compulsory coupledom” and the intersections of emotional/sexual lives with working conditions through gender and sexualities (Gusmano, 2019a, 2019b). Whereas the first approach creates a safer space for the community they do not push for political demands (e.g., partnering recognition, positive visibility, welfare protection and avoiding loosing children while divorcing due to their relationships) although they would support it, the radical approach carries more demands while at the same time creating non-normative networks of care “beyond kinship and love” (Gusmano, 2019b).

Although LGBT + and queer people might negotiate fidelity within heteronormative lens or more critical ones, the normative pressure of proving to be “good homosexuals” (Klesse, 2007) might have taken its toll on marginalizing consensual non-monogamous experiences as “undesirable practices” when fighting to achieve equality (Moors et al., 2017; Sheff, 2011) and unrelated to LGBTQIA + issues in Italy (Gusmano, 2019b). For LGBTQIA + people, the spectre of being promiscuous is still alive and well, often leading to reduce the support from mainstream associations to non-monogamous queer people. Polyamorous groups in the U.S. have previously avoided civil claims for fear of repercussions on the request of egalitarian marriage (Aviram, 2008), while on other occasions LGBTQIA + activism has excluded CNM issues to avoid being perceived as the ones demanding multiple or open-marriage recognitions (Cardoso, 2014). Even within the Italian debate for civil unions, the “fear of promiscuity” emerged through the imperative of “fidelity,” since conservative and democratic parties agreed to remove the principle of “couple fidelity” for same-sex civil unions, whereas it is stated as mandatory within heterosexual marriage (Lasio & Serri, 2019). A political statement aimed to position same-sex unions as “B-class unions” and make salient the representation of non-heterosexual people as inherently promiscuous and incapable of lasting exclusive relationships (Klesse, 2007), although the lack of this requirement might positively affect some people in non-monogamous relationships (Palazzo, 2018).

From a sociopolitical standpoint, in the Italian context LGBTQ + subjectivities have constantly encountered delegitimization, difficulties and open hostility regarding their struggles for recognition and civil rights (Crowhurst & Bertone, 2012; Lavizzari & Prearo, 2019). At the time of research, there was no national Bi+ association on the Italian territory, as opposed to GLT nation associations. In recent years, Bi+ issues have been more visible in Italian local associations with specific Bi+ associations growing in number and representations within queer spaces. The lack of a Bi+ visibility in Italy reflects the historical and erasing indifference within LG spaces (Angelides, 2001): national gay and lesbian association are reluctant to expose themselves on these issues, avoid promoting Bi+ events although they present themselves as LGBT + associations, biphobia and erasure are common experience within them (Castro & Carnassale, 2019). Bi+ people constantly face the erasure and invisibility (Yoshino, 2000) of their orientation even in their intimate relationships, where they might receive pressure to adhere to monosexual identities (Castro & Carnassale, 2019; Gusmano, 2019b; Monro, 2015). To bisexually oriented people, relationships can be a source of stress or a protective factor against biphobia (Mereish et al., 2017; Spalding & Peplau, 1997) depending on the degree of acceptance of their partner/s. Therefore, for non-monogamous Bi+ individuals finding a safe and reassuring space to affirm their sexuality can protect them from the general negative outcomes of biphobia encountered in Italian LGT spaces (Braida, 2020; Gusmano, 2019b; Scandurra et al., 2020).

### Accounts of Masculinities and Heterosexuality

The naturalization of male desires and the production of masculinity from heterosexuality received a huge amount of attention during the last decade of Men’s Studies, nationally and internationally (Bertone & Camoletto, 2009; Farvid & Braun, 2014; Ward, 2015). Going beyond its institutional and hegemonic characteristics (Connell & Messerschmidt, 2005), recent approaches have deconstructed the normativity of heterosexuality by inquiring its assumed stability as a sexual orientation (Savin-Williams & Vrangalova, 2013), the biological essentialism regarding male sexual functioning (Bertone & Camoletto, 2009) and, worth noting, the dissatisfaction of some men when their sexuality is simplified to “mechanic movements and biology” (Janssen et al., 2008).

The fluidity and flexibility of sexual orientation (Diamond, 2008) also involves heterosexuality in both positive (Savin-Williams & Vrangalova, 2013) and negative (Ward, 2015) expressions. Heterosexual men can experience same-gender attractions as part of their own desires and not by employing overmasculine discourse repertoires or practices related to “hazing” (Ward, 2015).

Deconstructing heterosexuality is hindered by the historical construction of heterosexuality as essential in hegemonic discourses to preserve normative structures of society (Hubbard & Hegarty, 2014; Ward, 2015). In his reflections, Connell (1995) positions hegemonic masculinity as intrinsically connected with women subordination and in opposition to both femininity—whether it is present in women or in men considered effeminate or feminine—and same-gender attractions, usually in the forms of male homosexuality. These hegemonic sense-making explain the omnipresent relationship between homophobia and the repression of non-heterosexuality as a despised “Otherness,” diametrically opposed to the hegemonic vision of masculinity (Ward, 2015). Within this frame, accounts of hegemonic masculinity do not provide a description of “real men,” a type of personality or a male “character” (Connell & Messerschmidt, 2005; Vandello & Bosson, 2013). It consists of an ideal or set of culturally available proscribed social norms symbolized both in discourses and in representations (Wetherell & Edley, 1999); it pervades both individual daily life and social activities among men and people of other genders. The hegemonic masculinity is presented almost as an ambitious goal far from the concrete everyday experiences or entirely possible, embodying a “fantastic” and “impossibility” aura in its nature (Kimmel, 2006; Vandello & Bosson, 2013) which generates anxiety and fear of losing the status of manhood (Vandello et al., 2008). The scripts of masculinity, arising from the essentialist imaginary of heterosexuality, shape how men position themselves to other gendered subjectivities in daily lives and within relationships, monogamous or not.

The present research inquired the stories and narratives of non-monogamous Bi+ and heterosexual men to offer an in-depth analysis of the meanings linked to consensual non-monogamies, sexualities, gender performances and how masculinity is negotiated in everyday life with relevant social actors (Pascoe, 2007; Wetherell & Edley, 1999) as “sexually oriented” men (Diamond, 2008). These two groups, Bi+ and heterosexual men, were chosen due to their different hegemonic positioning and shared desires towards women which can provide a useful comparison of accounts of masculinity. The study is part of a wider project on Bi+ orientation, heterosexuality and masculinity in Italy, a project informed by the critical reflections and findings on non-exclusive attractions and identities (Barker et al., 2012; Barker & Langdridge, 2008; Diamond, 2008, 2016; Rosenthal et al., 2012; Savin-Williams & Vrangalova, 2013). The theoretical frameworks of the paper merges narrative studies (Riessman, 1993), social constructionism (Gergen & Gergen, 1988) and sexual scripts (Simon & Gagnon, 1987) and aimed to answer the following research questions:

1. What models of masculinity are reproduced by men in non-monogamous relationships?
2. How desire towards multiple people and/or genders is conceptualized through men’s stories?

## Method

### Participants

Participants were 20 Italian men and two with a double nationality, currently in the Central or Northern part of Italy, half of them identified as Bi+ (attracted to more than one gender) and the other half heterosexual. Some Bi+ participants preferred to adopt a queer identity, while others adopted different labels across their lives from bisexual to pansexual and, sometimes, employing a wider definition of gay as a stigma management strategy (Schrimshaw et al., 2018). Age of participants ranged from 19 to 72 years old (M = 30; SD = 9) most of them falling between the age of 23–30. The study explored not just the stories of these men but also what types of practices and experiences were part of their personal backgrounds (Sell, 1997) regarding their relational practices (monogamous or non-monogamous) and three components of sexual orientation (Savin-Williams, 2006; Sell, 1997): attractions (fantasies, interest, sexual desire), identity (e.g., ace, Bi+ , demisexual, gay, pansex, heterosexual), sexual practices (e.g., search for sexual or intimate contact with one or more genders, sexual and emotional relationships, erotic fruition preferences and pornographic).

Most participants (80%) were non-religious, atheists or agnostics although they have been socialized as Catholics by their families, others followed a personal philosophy/spirituality closer to eastern religions than monotheistic western ones. These positions are plausible considering the main attitude towards non-heterosexuals, gender studies and LGBTQIA + people within most religious groups and the Catholic church in Italy (Lasio & Serri, 2019; Lavizzari & Prearo, 2019); only one Bi+ participant is actively involved in a Christian LGBT (but not QIA +) association.

Regarding the education of participants, only 10% had a tertiary education like a Ph.D. or a specialization course and the rest were similarly divided between having a bachelor/master’s degree (50%) or just a high school diploma (40%). Beside one student, all the heterosexual participants had, at the time of the interview, “stable” jobs (e.g., permanent contract) in the private sectors (e.g., researcher, engineers or techs, factory workers, tourist operators and one professional barista), whereas the Bi+ participants were 4 students, 3 had precarious or occasional jobs and 3 had stable jobs (one teacher and two office workers). Consistent with previous research (Gusmano, 2019a; Klesse, 2007), access to contractual power and stable incomes do affect the relationship lives of non-monogamous people, although this kind of analysis was not the focus of the present research, and studies suggest polyamorous people build relationships networks of support for their economic situation (Gusmano, 2019a).

Finally, the Bi+ and heterosexual groups of participants differed regarding their relationship styles of consensual non-monogamies. For Bi+ men polyamory is the most common relationship style (five out of ten), followed by three open relationships and two relational anarchists; their story accounts vary across their lives and these definitions describe their current, at the time of the interview, type of CNM they were actively engaged (6 out of ten) or would look for. Regarding the relationship styles of heterosexual participants, only two heterosexual participants engaged in polyamory or described their multiple relationships as also romantically involved, whereas all other heterosexual men were currently (3 out of 10) or previously involved (5 out of ten) in consensual open relationships after negotiating multiple attractions, as a rejection to monogamous norms or as a preferred relationship style. Demographics are given in Table 1.

**Table 1:** Demographics of participants

	Bi+ men	Heterosexual men	Total
	N = 10	N = 10	N = 20
Age (M ± SD)	31 ± 11.42	29 ± 4.3	30 ± 9
*Religious position*			
Atheist/agnostic/non-religious	71%	83%	76%
Catholic/Christian	12%	17%)	15%
Deist or personal spirituality	17%	0%	9%
*Education*			
Ph.D. or specialization degree	0%	1%	1%
Master’s degree or equivalent	17%	44%	29%
Bachelor’s degree or equivalent	30%	28%	29%
High school or professional diploma	50%	23%	40%

### The Narrative Approach

We could define narrative interviews as a way to “tell consistent stories in the course of an interaction” (Riessman, 1993) that allows us to inquire how and when “culture speaks for itself” through the history of the individual (Riessman, 1993, p. 5). The narrative process is also a performative act, which allows those who take part in it to perform relevant scripts (Simon & Gagnon, 1987) and providing accounts of individual, interpersonal and societal scripts within both the stories and the interaction with the interviewer; it gives authorship to those who tell their own stories and recognize themselves as both storytellers and part of the story, hence making narrative identity available (Murray, 2003). Studying narratives means focussing on the narrative process of storytelling to analyse the recursive telling, elaboration and interpretations of one own experiences (Emerson & Frosh, 2004).

As an interpretative method, the narrative interview privileges the perspective and the sense-making process of the narrator (Emerson & Frosh, 2004), the codes/metaphors used by them and their authorship. It also highlights the “particular moments” that Riessman (1993) defined as “insights”: moments of “understanding” or “awareness” of the participants themselves while they tell a story or as a “turning point” within the story identified by researcher. These insights are relevant indicators of the reflexive process that occurs in the encounter between the narrator and the listener, signifying that the story collection is not a passive process and that narrators are active and reflective agents (Emerson & Frosh, 2004).

Finally, within the paper the term stories it refers to the individual participant’s accounts and the set of stories as a corpus of data that allowed to analyse the narratives of masculinity and sexuality. Whereas by using the term narratives it refers to the analytical outcomes developed during the research from the storytelling and sense-making processes shared within the participants' stories (Emerson & Frosh, 2004).

### The Interview

The narrative interview used in this study was divided into 6 macro-themes to provide a thematic guideline for exploring relevant dimensions of sexuality and masculinity related to research questions (Riessman, 1993). The semi-structured interviews started with the same generative question (e.g., “Would you tell me a story about the last time you felt attracted to someone?”), which provides a story input that favours personal storytelling styles, and afterwards, the flow of the interviews was open and fluid; at the end of each interview, a set of structured questions inquired the components of sexual orientation and demographics. Clarifying the macro-themes and coding structure of the study is a relevant part in qualitative studies (Levitt et al., 2018), to promote the transparency and replicability of the research (Emerson & Frosh, 2004; Riessman, 1993). Following these reflections, the interview explored the following thematic dimensions:

1. Stories of interpersonal attraction.
2. Significant relationships and negotiations with social actors.
3. The construction of sexual orientation.
4. Bi+ or heterosexuality within societal networks.
5. Signifying masculinity.
6. Components of sexual orientation.

The interviews lasted from a minimum of 60 min up to a maximum of 135 min (mean duration: 95 min); no participant requested to interrupt the interview or expressed discomfort during the story collection process and further material on the study’s themes was provided afterwards on participant’s requests. The interviews were conducted face to face in a quiet and private place or online by using Skype; the audio of the interviews was recorded in both cases using a recorder or a recording software (Mp3 Skype Recorder). In the final parts of the interview, a change of rhythm by the researcher clarified a shift from the narrative focus on life stories to from more informative and direct questions about their attractions, identity, practices and sexual actions.

### Coding and Analysis

The stories collected through the narrative interviews were transcribed, double-checked and codified using the software Nvivo 12 and through a set of codes developed by integrating the theoretical framework of the researcher—like sexual scripts (Simon & Gagnon, 1987) and imaginaries (Wetherell & Edley, 1999) with findings from previous studies (Emerson & Frosh, 2004). The codes were organized in 3 hierarchical levels of analysis: structural codes, content codes and analytical codes. The first level of analysis included macro codes to identify the structural areas of interest like stories of attractions and sexual desire, masculinity or the relationships between genders. The second level of content codes were more specific and “captured” both topics of theoretical interest (e.g., discourses about non-monogamous relationships or coming-out) and potential meaningful constructions (e.g., if one participant discuss their sexuality as essential, genetical, socially learned or fluid). The analytical codes concerned the identification of transversal narratives in the participants' stories, aiming to identify the central nuclei of the narrative. The intersections between different analytical codes generated specific narrative themes, some of these are presented in the Results section: “Signifying Non-Monogamous Relationships,” “Signifying Identity Through Relationships,” “Imaginaries of Masculinity,” “Masculinity as caring partners,” “Masculinity Beyond Categories.” Codes are presented in Table 2.

**Table 2:** Codes employed during analysis and interpretation

Structural codes	Content codes	Analytical codes
Bisexuality and non-exclusivity Discourses and stories related to attractions to more than one gender, bisexuality, pansexuality and Bi+ identities	Care, Action and support Agency, Coping Strategies, Identities (e.g., related to “who I am”), Health and well-being, Negative feelings, Positive feelings, Professional support, Support by others	Becoming a sexually oriented man
Producing heterosexualities Discourse about straightness, attractions between man and women, acting heterosexual	Interconnecting codes Insight Inspirations Reflexivity Toxicity in relationships	Challenging scripts vs enforcing scripts
Masculinity and “being a man” To code discourses about masculinities, manhood, homosociality and performances of male gender	Non-heterosexualities and relationship styles Biphobia, Coming-out, Sexual fluidity and flexibility, Invisibility and erasure, Monogamy and non-monogamies, Non-heterosexualities passing, Prejudices, micro-aggressions and internalized stigma	Embracing uncertainty vs increasing rigidity within relationships
Gender issues To code discourses about gender, performances of gender, relationships between genders, normativity and roles	Performances of gender and body Charisma, Body and body practices, Drugs and substances, Aesthetics, Emotions, Masculinity, Gender models, Performances of gender and sexual Male politics, Policies, Sex and/or gender Sexuality or having sex Manhood	Enhancing Labels vs Going beyond Categories
Thoughts and theories of the sexual To code discourses and conceptualizations about the sexual, sexual orientation, identity, practices and relationships	Relevant networks Friends, Love and Intimacy, Communication and sharing, Relationship risks and difficulties, Families and parents, Flirting, Parenthood and kids, Jobs and working, Partners and meta-partners Religiosity and spirituality Society community and norms, Spaces	Constructing sexual orientation as Flexible vs Essential
Stories of attractions To code stories and extracts about attractions, desire, fantasies, having sexual encounters with actual actors	Imaginaries and scripting Expectations, Representations and imaginary, Real or anticipated reactions, Examples, cases, metaphors, Information or knowledge, Normal/Normativity and standards, Scripting, Explanations (biological) Explanations(evolutionary) Explanations (societal) Explanations (relational)	Precarious and reflexive masculinities

## Reflexivity

Adopting a reflexivity approach on one's personal assumptions has been a fundamental step in the research process and has been carried out through comparison with research notes, feedbacks of community experts of multiple sexual orientations and genders (Barker et al., 2012). Still, having conducted the interviews as a cisgender Bi+ man has plausibly elicited a certain type of relationships between the author and the participants (Barker et al., 2012; Levitt et al., 2018); discussing masculinities between men, or presenting as men, also has different implications, compared to other gendered subjectivities. Both myself and participants might have performed their gender scripts in a less hegemonic way, considering my positioning and their expectations of being evaluated; overall, discussing masculinity when interacting with femininity or fluid identities has different outcomes and performances. As informed by intersectional approaches (Cho et al., 2013), participants’ stories might have been told and/or interpreted, differently if conducted by another individual of a diverse gender, colour, sexual orientation, ability and class.

The study was part of a Ph.D. project on male sexualities and masculinities, exploring fluidity and non-exclusivity in men’s sexual desire, relationships and identities was the main drive of the research. Since feminists understanding of gender and sexuality are greatly beneficial to men’s studies, I followed a critical psychosocial perspective—informed by intersectional studies (Cho et al., 2013) and feminist activism—which allowed me to deconstruct my own forms of masculinity before understanding the ones of other men, queer or heterosexual. Having an activist background on CNM, bisexualities and masculinity required plenty critically informed reflections (Barker et al., 2012) to avoid celebratory positions of non-exclusive sexualities, consensual non-monogamies and masculinities. Hegemonic discourses and conflicts are part of the CNM community (Klesse, 2007; Sheff, 2006), and taking part online CNM spaces I could also observe how toxic relationships scripts involve both active CNM people and monogamous ones.

Within my research framework, I approach the sexual as a fluid continuum that rejects the binary assumptions of sexuality—as only female/male, hetero/homosexual—reinforcing heteronormativity, monosexuality and essentialism (Barker & Langdridge, 2008; Eisner, 2013; Yoshino, 2000). Participants of the present research were fully aware and informed of the aim of the research and the author’s background on masculinity, bisexualities and feminist studies, which have been often discussed or openly challenged by some participants (Klesse, 2007), mostly the heterosexual ones (Edley & Wetherell, 2001). While a deeper level of trust was achieved with the non-monogamous Bi+ participants, knowing that the interviewer is a Bi+ activist–researcher who conducts informative events on Bi+ sexualities and CNM. On the other hand, some heterosexual men were wary of feminism as a political stance that could help men or their masculinity (Edley & Wetherell, 2001), and this might be due to the availability of online pervasive discourses against feminism and “social justice warriors” (Coston & Kimmel, 2012). To build a trustworthy relationship, the author clarified and engaged in active listening and reassured that the interview is non-judgemental space, regardless of their identity and/or political positions on gender studies, feminism and men sexualities—which required self-analysis and expertise with manosphere arguments (Coston & Kimmel, 2012), a mandatory step for recognizing their storytelling authorship (Emerson & Frosh, 2004) and access potential hegemonic scripts of non-monogamous men (Sheff, 2006).

## Results

Participants engaged in active storytelling by discussing, and often challenging, the very terms employed by the researcher on defining bisexuality, heterosexuality, non-monogamy and manhood as labels that “successfully capture” their experiences and the labels themselves (Klesse, 2007). For example, although all Bi+ participants agreed on defining their sexual orientation as “bisexual”—or within the broader Bi+ definition of “attractions to more than one gender”—many felt the use of such labels for their own sexuality as a simplification or a convenient choice to easily clarify to others whose people they feel attraction to or not (Barker et al., 2012; Galupo et al., 2017). Of the 10 Bi+ participants, 5 men do use the term bisexual in a colloquial way to define themselves to others although they see themselves more as queer or unlabelled, following political preference, a rejection of labels or for a radical refusal of the gender binarism and the dichotomy of sexuality (Eisner, 2013); the term heterosexual was challenged only by those heterosexual participants that reported non-exclusive attractions. Their sexual encounters or desire towards men’s bodies was not signified as impacting their “whole identity” (Savin-Williams & Vrangalova, 2013) but as a potentiality to a wider range of experiences, sexual and intimate, they opted to “Enhanced their Labels” since they do not have constant attractions to more than one gender.

Clarifying diverse explanations of sexual orientation and gender was one of the aims of the study. To inquire these constructions the interview contained two generative and “provocative” questions: “Can you tell me about when you became bisexual/heterosexual?” and “Can you tell me the story of when you became a man?” These questions might have been perceived as provocative due to the sensitive nature of the ontology of sexual orientation, which might be felt as erasure for LGBTQIA + individuals (Diamond, 2012) or challenging the normativity of heterosexuality (Ward, 2015); they were not uncritically accepted by participants. There were frequent request of clarification almost to test the basis on what kind of explanation the question “demanded”; other times the generative questions have been openly challenged (e.g., “I don't think you can become one”) preferring other “scripts” (Gagnon et al., 1999; Simon & Gagnon, 1987) to interpret and signify their sexual orientation and/or gender.

### Signifying Open Relationship

Consensual non-monogamies (Veaux & Rickert, 2014) are valid relationship styles which take many forms under the CNM umbrella (Barker, 2010). According to Peabody (1982), the pillars of consensual non-monogamies consist of: honesty communication, equal power in relationships, privacy, trust and more personal and interpersonal development than monogamous relationships. Growth and personal development are significant outcomes in these relational styles, so much to be principles to follow (Mitchell et al., 2014), a motivation for engaging in CNM relationships and outcomes to be achieved within them (Aguilar, 2013).

Considering the diverse forms of CNM (e.g., polyamory, open relationship, relational anarchy, etc.), it was not surprising to see that open relationships are the most common form of non-monogamy, especially for heterosexual participants since they often navigate more dominant scripts (Farvid & Braun, 2014; Simon & Gagnon, 1987). To heterosexual participants, shifting from monogamy to consensual non-monogamous agreements was often signified as plausible way to manage attractions to more people at the same time (Deri, 2011) and rarely an approach to criticize the hegemony of monogamy (Ziegler et al., 2014). These attractions were essentially related to their sexual drive at an individual level, and only a few linked it to the biological functioning of male sexuality (Bertone & Ferrero-Camoletto, 2009); on the other hand, Bi+ individuals signified their desires as linked to their sexual orientation more than their gender, implying a substantial “need” of experiencing diverse attractions as part of them. Desires are sometimes signified as feelings “beyond control” which the individual must manage and negotiate to avoid hurting the relationship, the following extract provide an example:

“precisely because this (attractions) happens a lot I immediately talked about them to my girlfriend…since I know that these attractions happen all the time…it’s something that I do not control so there was not a lot to say about it. Let's say we decided to have a relationship where…you can approach another person we are attracted to until these…“things” do not affect us. Does not interfere in our relationship.” Homer (Heterosexual man, open relationship, 23 years old).

Although he actively clarified the principles underlying their agreement and what situations could be framed as “problematic” and motives to “take a step back” (e.g., wanting a primary romantic relationships with another person), by conceptualizing his desires as essential Homer creates a hierarchy of needs to be met by his partners. At the time of the interview, Homer and his primary woman partner were already seeing other people, while maintaining a long-distance relationship for 3 years. Within his story, he reflects on the assumption of monogamy as more socially scripted and proscribed (Simon & Gagnon, 1987) compared to his essential conceptualization of desire (Hubbard & Hegarty, 2014), as “something he cannot control,” which plausibly drives challenging monogamy within their relationships:

So, we have come to understand that being together is something that has been spoiled by the culture in which we live, so there is this taboo…of being monogamous or, for example, the fact that if you are in a relationship you must feel guilty…if you feel attracted to others…and therefore no one can absolutely approach anyone else without having to feel almost guilty for doing so…but we are both children of a cultural heritage that we do not share…therefore we said “let's see what we can do…to avoid to suppress our feelings,” without, and this is a standpoint for us, hurting the other person” Homer (Heterosexual man, open relationship, 23 years old).

Negotiating consensual non-monogamies within other-gender heterosexual relationships was, for Homer, part of a process of deconstructing attractions outside of coupledom and their social consequences from relevant groups (Barker, 2005; Wosick-Corea, 2010). In his story emerges a will to “Challenging Scripts” rather than conforming to them completely; in fact, hegemony still lingered when asked about polyamory, and if it was a plausible solution, he reported that it was not something for him, distinguishing between sex with multiple people and romantic investment (Sheff, 2006).

Having access to non-monogamous communities is a major step towards acceptance and challenging monogamous assumptions, since sharing experiences, feelings and strategies for negotiating relationships is a form of social support for non-monogamous people (Bauer, 2014). The lack of information about online or offline spaces for CNM increases the risks of potential conflicts with partners and having more emotional labour if only one partner is non-monogamous (Klesse, 2007). It seems that heterosexual men who engage in open relationships less frequently participate or engage with relevant CNM networks, which plausible makes it harder for these heterosexual men in open relationships who lacks these feedbacks to follow ethics of care (Conley et al., 2013; Gusmano, 2019a). The story from Tennents discuss the rising of jealousy in his open relationships and the challenges of overcoming it without discussing it with anyone, refusing to look for help was counterproductive to his masculine imaginary and his assumption that open relationships are easy. Not looking for support was his first choice for managing an open relationship not anticipating that jeolusy might arise even with unstructured agreements:

“After a year…that jealousy began…rising again and again, whereas being in an open relationship the concept of jealousy must be erased…as time passes it goes away little by little while other feelings arise…[How one can negotiate an open relationship?] The open relationship we have negotiated…well it is not that we have ever talked about it…we discussed about this lightness we wanted and…since it (the relationship) was born by chance.”

Tennents (Heterosexual man, open relationship, 27 years old)

In his account, Tennents reports how he had to recognize a growing feeling of jealousy towards his main partner in a long-term open relationships, a feeling that in his opinion would be erased over time instead of openly discussed (Deri, 2011). Tennents never heard of groups to support CNM people or thought they might be worth looking for. Although he has engaged in open relationship for 5 years he selectively avoid emotional disclosure to partners, believing that total acceptance and disclosure to a parter was unachievable in his relationships. Moreover, in signifying "lightness" as the opposite of the need to communicate one’s needs some participants laid the ground for the rising of jealousy and insecurity:

“No, we have not talked about it and we could say that…it is a bit of a difficult subject for me…seeing that the whole relationship was focused on this lightness…and we’ve always said that as soon as we see that the situation is getting out of hand we should talk about it…it's hard to talk about it when you think it could end or change…even the slightest change in my opinion.”

Tennents (Heterosexual man, open relationship, 27 years old).

Although striving for more lightness and less rigidity is cherished in these relationships, the story of Tennets and others does resonate more with the anxieties of failing one's partner/s and the shared agreements; looking for lightness as a relationship strategy might imply more control than freedom (Sheff, 2006). Although both Homer and Tennents engage in the deconstructions of mononormativity, they still linger to degrees of hegemony regarding how these relationships must be preserved. This might be due to the lack of feedback and not looking for support in CNM communities, not discussing agreements and CNM issues affecting their relationships’ expectations and outcomes (Conley et al., 2013). Open relationships, like any other CNM, have their challenges and needs, but without community feedback their representation is scripted as “simple,” “light,” and “for everyone,” which lead to unpleasant negotiations and breakups.

### Signifying Identity Through Relationships

In contrast to heterosexual participants, for the Bi+ participants in the study their non-monogamous relationships allowed them to explore and signify their bisexual attractions to more than one gender (Barker, 2010; Manley et al., 2015) and finding more Bi+ positivity within CNM spaces since they’ve already deconstructed the assumption of mononormativity (Gusmano, 2019a). Moreover, challenging and negotiating labels is also part of many non-monogamous individuals, to which the very idea of sexual labels or definitions for their feelings/experiences does not grasp their everyday lives (Eisner, 2013; Klesse, 2007). The narrative themes of “Going Beyond Labels” and “Embrace Uncertainty” were relevant in the story of Foglia (“Leaf” in Italian), which identifies as relational anarchist and clarifies how for him relationships are person-specific (Diamond, 2008) whether they are with women (most of the time) or with masculine men:

“First, I don't recognize myself with the bisexual label…I recognize myself in an idea of love and not in definitions about it…any relationship they (relational anarchists) have has the name of the person they have a relationship with…for example with you A. I have a relationship called Leaf-A. whereas…if I have a relationship with Francesca that relationship has her name. In the end is a way to narrate myself…I am a human form that relates to other human forms.”

Leaf (Bi+ man, relational anarchist, 45 years old).

Within his story, Leaf discloses his definition of relational anarchy (Nordgren, 2006), which almost compels him to clarify how labels are too tight for describing his person-specific approach (Diamond, 2008); by recognizing that each relationship is unique, Leaf suggests that “people” should focus on finding a shared connection as human beings rather than gender. Still, he later clarifies that gender characteristics are quite relevant in his attractions especially those for men, since he prefers overly masculine or, at least, nonfeminine men; feminine men are labeled as “too stereotypical” to be attractive. This story provides an example of poly-hegemony where men can both transgress and enforce hegemonic power norms (Sheff, 2006) by following an hegemonic imaginary of “attraction to real men” and anti-femininity attitudes often found in the discourses of gay and Bi+ men (Connell & Messerschmidt, 2005; Klesse, 2007; Sheff, 2006). He construct the bisexual orientation (Barker et al., 2012; Sheets & Mohr, 2009) as “something” related to personal interactions and desires which might always arise for everyone; in his case, he retells the story of his first same-gender attraction at the age of six to position his attractions for men and women as essentially “always present” (Katz-Wise & Hyde, 2015):

Having safe spaces to perform and freely express your sexual and relationship orientation is fundamental for Bi+ individuals, which usually lack trust in heteronormative relationship and LGT spaces due to previous experiences of biphobia and erasure (Barker et al., 2012; Schrimshaw et al., 2018). Spaces/associations that do not comply with heteronormative assumptions are considered safer and welcoming for non-monogamous Bi+ individuals (Bauer, 2014). In his story, Enrico, a bisexual poly man, discussed how expressing attractions towards a potential new meta-partner, a bisexual woman, in a BDSM space was easier due to the interweaving of desires, practices and performances within these spaces. Feeling less anxiety and judgement in a safe space allowed them both to manage their attraction better than in the “outside world” (Deri, 2011):

“ they are called performances…because I actually did it in public (laughs). There were others who looked at us…so Giulio and Veronica found themselves buttnaked to be whipped my me…Giulio is so sweet, a friend for whom I felt this physical but sweet passionate moment…whereas for Veronica there was this heavy [emphasis] sexual attraction…she made me feel extremely sexually aroused.”

Enrico (Bisexual man, poly, 34 years old)

In the story of Enrico emerged the surprise of having again (after a few years of only dating men) meaningful attractions to women due to the fear of “missing out” from one gendered “world” and not being “bisexual enough.” A common theme in Bi+ accounts, since specific delegitimizing myths of bisexuality (Rodriguez Rust, 2002) have created the idea that bisexuality is always incomplete or lacking. The myth that “men can’t be bisexual” puts a heavy toll on the experiences and desires of Bi+ men (Castro & Carnassale, 2019; Pallotta-Chiarolli, 2016; Rosenthal et al., 2012) and how they perform their masculinity with significant others.

The Bi+ participants in this study considered their CNM relationships as a positive discovery and a way to reaffirm their identity as “Becoming a sexually oriented man,” rather than a necessity to manage sexual desire. Desires that were not signified as essential to their masculinity. To others, CNM allowed to explore feelings and imaginaries closely connected to their identities (Gusmano, 2019b), to fully express all the attractions to each gender they are attracted to:

“after (my) bisexuality I started to experiment mentally…thinking about new types of relationships…I approached polyamory and my sexual tastes changed too…meaning that if maybe before I used to look at pictures or videos with women…pornographic or not…now I definetely prefer seeing stuff with more people, male and female.”

Roxas (Bisexual/Queer man, poly, 24 years old)

To Roxas, sexual and relationships desires became interconnected for signifying and further exploring his sexuality, describing his masturbatory fantasies to more people as “much more meaningful” and “a different story” compared to single gender/person fantasies.

Although CNM can be a source of positive relationships outcomes, they do require emotional work, openness, responsiveness, time management and transparency within the relationship, respectful negotiations (Anapol, 2010; Barker, 2010) which might be demanding and difficult to achieve. Resisting hegemonic scripts of masculinity and power dynamics is not an easy process for both groups of participants (Sheff, 2006), aspiring to new values increases the risks of conflict within the relationships, especially if one of the partners has only recently engaged in consensual non-monogamies. Roxas is a Bi+ who came out as bisexual over the last 2 years and at the time of the interview he engaged in a polyamorous relationship with his partner Giulia, also bisexual and poly, where he found a great amount of support and acceptance for his bisexual orientation (Feinstein et al., 2016) but still struggled concerning the non-monogamous agreements and boundaries when it concerns other men (Sheff, 2006). In the following extract, Roxas discusses the potential inclusion of a meta-partner that they both knew, to which Giulia is attracted but he is unsure about including him in their poly relationship; sharing the worries and setting boundaries is signified as a step-by-step journey of coming to terms with personal feelings and preferences:

“Sometimes I just bang my head against a wall I already told you about Francesco, this person that I might meet today and It might happen that we will sleep together and I had to work a lot on it […] There were moments sometimes when I wanted Giulia to go with him because I wanted to know…if I know that I do not feel good about it we can close it. Then I realized that was a wrong on my part and I must give them time…is something I have rights on, I can immediately voice myself…and that we can do this together instead of thinking…“if I don't do it then she runs away or maybe she does it anyway”…or even the idea that “I have to do everything right.” That’s how I was living it…whereas now I'm trying set my rights and to understand that they are preferences.”

Roxas (Bisexual/Queer man, poly, 24 years old).

Resisting hegemonies is a continuous emotional work that requires to accept failures, and sometimes hegemonic complicity, as part of the process; some of these CNM men had to “Challenging scripts” they were socialized up to avoid hegemony (Sheff, 2006). The pay-off is having a supportive network of partners and meta-partners that accepts them; for Bi+ men, it can lead to a buffer against negative outcomes of biphobia (Feinstein et al., 2016; Mereish et al., 2017).

### Imaginaries of Masculinity

The hegemonic ideologies of gender relationships maintain, legitimize and naturalize the interests of the dominant group while, at the same time, actively marginalize the demands of other groups as subordinate identities (Connell & Messerschmidt, 2005; Wetherell & Edley, 1999). To understand how men can resignify or reimagine masculinity research has focussed on how diverse models of masculinity construct their relationships with accounts of femininity, same-sex desires and sexual prejudices.

Through the narrative analysis diverse imaginary of masculinity were constructed in relationships with hegemonic models, whether abiding with them or resisting to them. The main sense-making processes involved in the narratives could be positioned inside a continuum between the “Enhancing Labels” which is a resignification of masculinity through “reflexive” and non-hegemonic lens (Anderson & McCormack 2018) or by adopting an imaginary position that “Goes Beyond Categories” challenging the idea that there are masculine or feminine characteristics altogether in favour of “being a person.”

### Masculinity as Caring Partners

The imaginary of men, and manhood, as protective towards women is part of a representation of masculinity that positions men as the “stronger” gender both physically and psychologically (Day, 2001; Bertone & Ferrero-Camoletto, 2009), an argument historically employed to justify the patriarchal system of oppression that aims to control women’s autonomy in favour of male’s control (Connell & Messerschmidt, 2005; Ziegler et al., 2014). Due to its wide availability, this narrative repertoire is usually perpetrated by sustaining that men are more rational, competent and reliable by presenting biological or evolutionary studies about gender difference (Rippon, 2019). Although this script has many hegemonic sides, within consensual—and plausibly non-consensual—CNM relationships there might be different stories and protagonists available for building scripts of “care” within other community context (Bauer, 2014). Principle of ethics of care provided within consensual non-monogamies and BSDM might facilitate negotiating the hegemonic repertoires of “protection” to “care.” The following extract by Benito—who is a mostly heterosexual man who reports multiple sexual attraction to men and engages in consensual open relationships and the kinky/BDSM community—reflects on positive aspects of his masculinity that intersects with his body type:

“Are there any characteristics of your idea of masculinity that you see as positive compared to others”?

Yeah, I don't know if it's something I do because I consider myself a man (enphasis) or because that’s what I do…it's the fact that I'm large and big, I am more inclined to the role of caregiver. Very often my affection to the people I fell in love with or to my friends is mostly based on being like a hen, let's call it like that…I'm the one who, if I see that they have a problem, moves to try to solve it…I think it's […] the “big guy” syndrome…you can be “big” as much as you want but if you're big just to intimidate others you are fucking useless, if you're big it's because you defend others. Hence, masculinity…manhood can also find a positive meaning in the social role of man as a warrior, not a warrior as the one that wages war but as the one that defends…let's not call it a paladin (like a knight) because nobody likes the idea of the “good guy.”

Benito (Heterosexual man, non-monogamous, mostly heterosexual, 33yo)

By discussing the role of caregiver to lovers and friends, Benito reflects on how his body type has been a part of his personal development and views of masculinity as a scripted role (Simon & Gagnon, 1987), which he defines from being a “big guy.” He resignify masculinity “Enhancing the Label” by not relying on being aggressive and “wage war” but on following a path of “care” and “responsibility” towards others and oneself. When asked to tell the story of “becoming a man” he provided a first brief story of his first sexual encounter with a woman but felt unsatisfied with this account of masculinity (Wetherell & Edley, 1999); afterwards, he had an “insight” (Riessman, 1993) and he choose the theme of responsibility, a principle he recognized after the loss of his father, merging it with the ideals of consent and care due to his kinkster identity.

Although this story remains within the heroic imaginary, which is linked to typical masculine role of being “action oriented” (Wetherell & Edley, 1999), by situating through ethical principles (Anapol, 2010) it facilitated the deconstruction of “protective but not caring.” This view resonates with available frameworks for BDSM negotiation like the 4Cs (Caring, Communication, Consent and Caution) introduced by Williams et al. (2014) to describe care as a central pillar of BDSM practices. The two non-exclusive heterosexuals were both involved into BDSM practices, open relationships and were comfortable with same-gender sexual acts, regarded as “practices” to try after discovering anal masturbation.

The principle of care might also be followed in overly dominant approach that might oppose the principles of equality and care (Deri, 2011; Gusmano, 2019b) even if emerging within “caring” discourses:

“Sweetness but it is also a dominant person…so my masculinity It's pretty obvious…you heard that I had things that I wanted things that I didn't want…but whatever the situation it’s always in warm welcoming way. […]…for me the idea of ​​a new masculine…It’s that of a masculine that is very…soft even though I identify a lot with a paternal figure in a sense…regardless of that because I have no children and therefore I cannot…I talk about it.”

Leaf (Bisexual man, relational anarchist, 44 years old).

In these two extracts, the participant discusses a different view of masculinity from Benito, as masculinity is performed with more dominance and control rather than equality and autonomy (Anapol, 2010). CNM are not exempt from dynamics of power and imbalance, as Cascais and Cardoso (2013) have shown, and some narrative repertoires might often imbued with benevolent sexism and patriarchal assumptions related to possession and power; they suggest this mostly happens in the discourses of “new” people to non-monogamies, therefore in the passage from monogamous relations to non- monogamous. Although there are narrative repertoires of “care” and “mentoring” within participants’ accounts, there is also the risk that caring becomes paternalistic (Ziegler et al., 2014). Relationships with monogamous people, even for occasional sex, might also be problematic:

I must explain all my philosophy again and then people start to love me…now look I do not want to seem excessive…but let me make a digression I want you to listen to, you I have a problem of…of attachment, meaning that…when people find out how you are then you create a relationship. They become a bit addicted…and so they feel bad if they are not there (for them). You create a little bit of this dynamic here, this is what I felt.”

Leaf (Bisexual man, relational anarchist, 44 years old).

Finally, in Leaf’s story he discuss a risk in endorsing a personal approach to relationships which defines as “addictive” and “charming” to those who do not know his approach to relational anarchy; it might be that some principle of non-monogamies might encounter a kind of fetishization by monogamous people (Klesse, 2007) that does not promote autonomy.

### Reflexivity Beyond Categories

Positive models of masculinity have been discussed by other psychosocial approaches on how men could resignify or abandon traditional/hegemonic aspects of masculinity (Connell & Messerschmidt, 2005) thanks to a progressive direction of general society (Anderson & McCormack, 2018). Others suggest that challenging dominant masculinities is still an ongoing process affected by both interpersonal experiences and historical construction of sexuality (Hubbard & Hegarty, 2014; Ward, 2015; Wilson et al., 2010) and that some, apparently, progressive sexual actions (e.g., like sex between heterosexual guys) or critics of overmasculinity (e.g., despising physical strength for argumentative strength) might just be a reskinning of hegemonic practices (Ward, 2015) or dominant male discourses (Coston & Kimmel, 2012). Still, reflexive and inclusive models (Anderson, 2018) of masculinity are present and available within male narratives and to the men participants of this study. To position these narratives, two extracts from a Bi+ and a heterosexual man discuss whether it is truly necessary to become a “man” and the effort to refuse conforming to hegemonic models by “going beyond categories”:

“Thanks to my studies…I throw all of these pressures out of the window, because I mustn’t necessarily become a man. What does it mean in the first place? and, I do not even care about being a man…because a man is just a person and does not rely on these stuff but other values. [What kind of values?] Honesty, being a good person who cares about others. For me it is very important the concept of empathy, something that I noticed you do not need it to be a man. But for me it is important.” Roxas (Bisexual/queer man, poly, 25 years old).

In his account, Roxas discussed how expectations of “traditional masculinity” (Connell, 1995) imbues everyday life from the competition in his football team (where he is still closeted as Bi+) to the expectation of “being a man” with his family; he regards empathy as a characteristic “rejected” by traditional masculinity, since it is seen as an “essentially feminine trait.”

Reflexive accounts concern the personal and societal assumptions on their narrative identity (Murray, 2003) and everyday lives (Wetherell & Edley, 1999). They are not necessarily less hegemonic than resignification of masculinity, there are normativities also in queer repertoires, yet they inform us on the expectations and gendered relationships we can find in relationships, whether they are monogamous or not. Resisting and deconstructing male privilege requires a huge amount of work and self-reflection (Klesse, 2007; Sheff, 2006). An interesting example is provided by Homer, as suggest that a path to reflexivity lies through vulnerability and openness, on being able to expose oneself freely within one’s relationships with others, romantic or not, which is regarded as more “difficult” to achieve within male relationships and spaces (Anderson & McCormack, 2018; Bertone & Ferrero-Camoletto, 2009). To challenge hegemonic discourse the central theme of “evaluation” and “shame” proved relevant within male CNM narratives, a normativity that might be resisted through non-judgmental relationships and spaces:

[Were there any insights for your reflections?]

that we originate from other men. And I believe that another option is also to find a person with whom we can talk or be ashamed about ourselves…I think that shame slowly disappears or that it’s reduced anyway. Finding someone to be more open to…it seems like a trivial thing, like finding a best friend. In fact, I find there is also a distinction that I see in friendship relationship” Homer (Heterosexual man, open relationship, 23 years old).

In this excerpt, Homer expresses his imaginary position of reflexive masculinity (Anderson & McCormack, 2018) to hegemonic discourses, that male difficulties and challenges, “derive” from other men and that they are connected to personal history as well as to social contexts. The theme of shame and guilt are present transversally and are connected to the pressure to adhere to male hegemonic models (Connell, 1995; Vandello et al., 2008). Setting the requirements of the “true man” as a concretely unattainable model (Kimmel, 2006) while, at the same time, categorizing those who fails achieving it as “inadequate” or “failures” explains many male experiences under patriarchal norms. Within these masculinities, hegemony is maintained in ciclical performances of prevarication and complicity between men who follow the masculine ideal at the expenses of other genders, other men, and themselves (Bertone & Ferrero-Camoletto, 2009; Farvid & Braun, 2014; Vandello et al., 2008). Shame and guilt are tools of social control to maintain this hegemony, discouraging people from moving away from other models and making it difficult to make new ones (Klesse, 2007; Ward, 2015). Indeed, shaming men who deconstruct masculinity and resignifying privilege as victimhood has been a recurrent strategy for antifeminist and supremacist groups in the manopshere (Coston & Kimmel, 2012). As a final remark, Homer positions himself using an heroic imaginary that still navigates the normative discourses of masculinity (Farvid & Braun, 2014), but offers us an insight into how the narrative repertoires can make sense and overcome hegemonic pressure to repress man’s feelings through shame:

“Precisely because it is difficult for me to do it…it should be taken into consideration as a way of overcoming a certain type of manhood…trying to talk to male people about personal things” Homer (Heterosexual man, open relationship, 23 years old).

## Discussion

The present research aimed to investigate the stories of attractions of non-monogamous Bi+ and heterosexual men, in the Italian context, by focussing on their intersections between gender and sexual orientation. By focussing on these two groups, the study addressed the growing demands for the recognition of Bi+ and CNM issues within social sciences and the LGBTQIA + community, which has historically erased or made invisible those attracted to more than one gender and/or person (Angelides, 2001; Gusmano, 2019b; Rosenthal et al., 2012). Also, the interviews explored the imaginary positions of masculinities available to these non-monogamous men, to understand if and how they can resist the pressure of adhering to masculine norms while, at the same time, challenging mononormativity.

The stories analysed in the present research are consistent with recent studies on non-exclusive sexuality (Diamond, 2016; Diamond et al., 2017; Galupo et al., 2017; Savin-Williams & Vrangalova, 2013) and non-monogamies (Bauer, 2014; Gusmano, 2019b; Moors et al., 2017) which shows how non-exclusive desires and relationships are valid and linked to positive outcomes in life. Stories about relationships, whether they are monogamous or not, are affected by how we conceptualize orientation, desire and imaginaries of gender (Katz-Wise & Hyde, 2015; Wetherell & Edley, 1999). Signifying desires to more than one person through shared repertoires of relevant CNM networks was an effective strategy for Bi+ participants who navigated diverse forms of CNM (Gusmano, 2019b). Heterosexual participants lacked this network support since they, almost totally, engaged in open relationship kept privately; this strategy increased the risk of underestimating the challenges of non-monogamies by pursuing an initial idea of “relationship lightness”—which afterward led to huge amounts of negotiations or to breakups (Sheff, 2006). Overall, most CNM participants resisted hegemonic models of masculinity (Sheff, 2006), but heterosexual men reported higher difficulties in finding supportive and non-judgemental homosocial interactions (Connell & Messerschmidt, 2005).

The research also explored diverse performances of bisexualities, from the difficulties faced by Bi+ participants due to erasure, invisibility and biphobia (Anderson & McCormack, 2016; Ochs, 1996) but also the supportive potential of acceptance and relationships for bisexual orientations in CNM spaces. Moreover, consistently with research on mostly heterosexual (Savin-Williams & Vrangalova, 2013), a minority of non-monogamous heterosexual men reported non-exclusive attractions towards other genders and body types, endorsing a more flexible view of sexuality and sexual practices.

Findings also showed how non-monogamous agreements and relationships are signified through different kind of stories and masculinities (Wetherell & Edley, 1999). Some more essentially related to attraction outside of coupledom as “normal” or “natural” which should be managed, whereas others reflected on the normativity of monogamy as a social structure which should or must be challenged (Braida, 2020; Eisner, 2013). These positions are more a matter of degree than kind, as some participants recognized both as part of their story (e.g., these attractions are part of me, and monogamy is socially enforced rather than natural). The study does not argue that one position is “better” than another, but it suggests that managing consensual multiple relationships (Deri, 2011; Gusmano, 2019b) passes through these stories and addressing them might prove resourceful to non-monogamous people.

### Limitations

Due to its small sample, the following research does not claim to be exhaustive or representative of all men’s accounts of CNM, even if generalizing findings (Levitt et al., 2018) is relevant to sexual studies. We can therefore expect different understandings and definitions of masculinity, sexuality and consensual non-monogamies all equally valid (Barker et al., 2012; Braida, 2020). Masculinity is not only produced, handed down and built by the masculine genders (Connell & Messerschmidt, 2005; Farvid & Braun, 2014), hence lacking the voices of non-binary people and women socially constructing masculinity and consensual non-monogamies is a limit of the study (Bertone & Camoletto, 2009; Gusmano, 2019a). The demographic of the, almost totally, young adults sample reflects the Italian context but lacks diversity in colour, migratory status, ability, class, marital/relationships status and having children; since house property and negotiating families are a large part of CNM issues (Klesse, 2007) and masculinity is reproduced also in family contexts and via parenthood, unfortunately only one older Bi+ participant has children and was still coming out to his family. Finally, the two groups differed in their forms of CNM therefore to further understand how they further negotiate other CNM relationship beside open relationships is a limit of the study.

### Conclusions

The present research project aimed to voice and represent a plurality of stories of non-monogamous Bi+ and heterosexual men in the Italian context, emphasizing the available narratives to signify masculinity, sexual orientation and identities. Exploring the sense-making process within the stories enhances our understanding of identity and relationship negotiations (Bruner, 1990; Gergen & Gergen, 1988; Ritchie & Barker, 2006), from how we signify a Bi+ orientation (Barker et al., 2012; Castro & Carnassale, 2019) and the variability of desires (Diamond, 2008) to the production of heterosexual masculinity (Ward, 2015).

Using generative questions to stimulate the creation of stories proved effective for inquiring these intersections. Relationships are meaningful stories that we tell together and shape through our imaginary positions; each one offers insights into what narrative repertoires are available and the degree of hegemony men are performing or resisting (Sheff, 2006; Wetherell & Edley, 1999; Wilson et al., 2010). Diverse imaginaries of masculinities can support and create new stories within relationships, the present study suggest that signifying the idea of “protection” as “Care” (Gusmano, 2019b) might enhance positive and reflexive masculinities for those men who “Enhanced the Label” of masculinity resisting hegemonic models. Expanding the principle of “Communication” (Williams et al., 2014) was most useful for the Bi+ men who constructed gender as “Going Beyond Categories,” since they had to negotiate not only CNM relationship agreements but their desires to more than one gender as a way to achieve a Bi+ and/or queer identity. Encountering and developing CNM relationships and desires strengthened the principle of “Consent” (Williams et al., 2014) by challenging the predatory model of masculinity which sees men as a biologically driven sex machine (Bertone & Ferrero-Camoletto, 2009).

Narratives of non-monogamous Bi+ and heterosexual men showed how CNM agreements might arise from diverse conceptualizations of desires, feelings, relationship experiences or radical positioning towards normative assumptions. Available male models of masculinity are negotiated, navigated and, sometimes, resisted when encountering non-normative scripts of gender, sexuality and relationships; more psychological studies should address and recognize the complexities of masculinities to intercept the calls for more reflexivity within their gender to problematize it (Anderson & McCormack, 2016; Farvid & Braun, 2014; Ferrero Camoletto & Bertone, 2010; Ward, 2015).

Finally, it is worth noting that participants’ stories and experiences problematized both the practices and the meanings (Emerson & Frosh, 2004) linked to their identities and CNM relationships, conceptualizing them more like an ongoing process than a fixed outcome (Gusmano, 2019a). Like coming-outs, managing CNM ethics and boundaries is a circular and continuous event (Gusmano, 2019b) that becomes part of the participants’ story and a sexual script in their own way (Ritchie & Barker, 2006; Simon & Gagnon, 1987). It is always possible to tell and create different stories about relationships even within contexts of hegemonic narratives through diversity and reflexivity. By giving voice to these stories we have the potential of making them available to those who, during their “character development,” look for different stories to make “reality” more intelligible and less lonely.
